# Bioremediation Potential of Leaf Endophytic Fungi in *Allium ampeloprasum* and *Brassica oleracea* var. *capitata*

**DOI:** 10.3390/jof12040295

**Published:** 2026-04-20

**Authors:** Dayani Pavalakumar, Sagarika Kannangara, Nadeema Dharmasiri, Chamani Amarasekara, Lanka Undugoda, Kasun M. Thambugala, Jayantha Munasinghe, Sukanya Haituk, Ratchadawan Cheewangkoon

**Affiliations:** 1 Department of Biosystems Technology, Faculty of Technology, University of Sri Jayewardenepura, Homagama 10200, Sri Lanka; dayani.dx@gmail.com (D.P.); nadeed079@gmail.com (N.D.); 2 Department of Plant and Molecular Biology, Faculty of Science, University of Kelaniya, Kelaniya 11600, Sri Lanka; sagarikadpk@kln.ac.lk (S.K.); chamaniruwanthika@gmail.com (C.A.); 3 Genetics and Molecular Biology Unit, Faculty of Applied Sciences, University of Sri Jayewardenepura, Nugegoda 10250, Sri Lanka; kasun@sci.sjp.ac.lk; 4 Center for Biotechnology, Department of Zoology, University of Sri Jayewardenepura, Nugegoda 10250, Sri Lanka; 5 Center for Plant Materials and Herbal Product Research, Faculty of Applied Sciences, University of Sri Jayewardenepura, Nugegoda 10250, Sri Lanka; 6 Department of Mathematics, Faculty of Science, University of Kelaniya, Kelaniya 11600, Sri Lanka; munasing@kln.ac.lk; 7 Department of Entomology and Plant Pathology, Faculty of Agriculture, Chiang Mai University, Chiang Mai 50200, Thailand; sukanya.h@cmu.ac.th

**Keywords:** *Allium ampeloprasum*, bioremediation, *Brassica oleracea* var. *capitata*, endophytic fungi, HPLC, PAHs

## Abstract

Polycyclic aromatic hydrocarbons (PAHs) are toxic air pollutants mainly released through vehicular emissions and can accumulate on edible plants, posing health risks to humans. This study aimed to isolate and identify endophytic fungi from *Allium ampeloprasum* and *Brassica oleracea* var. *capitata*, which are widely cultivated along roadside areas in the upcountry region of Sri Lanka. Sampling sites included Nuwara Eliya town, Nanu Oya, St. Clair’s, and Meepilimana (control), where above-ground parts of the selected vegetables were collected in six replicates. Fungal isolates were obtained through surface sterilization, and their ability to degrade PAHs (naphthalene, phenanthrene, anthracene, and pyrene) was evaluated using plate assays, spectrophotometric analysis, and high-performance liquid chromatography (HPLC). Phyllosphere PAH concentrations were also measured using HPLC. It revealed significantly higher concentrations of all four PAHs in the phyllosphere of both vegetables at polluted sites, with the highest levels recorded in *A. ampeloprasum* from Nuwara Eliya town: naphthalene (145.92 ng/g), phenanthrene (97.67 ng/g), anthracene (88.71 ng/g), and pyrene (63.82 ng/g). Most endophytic fungal strains isolated from both vegetables were able to grow on Bacto Bushnell–Haas (BBH) medium supplemented with PAHs, producing colonies exceeding 20 mm in diameter. Spectrophotometric analysis showed that *Fusarium liriodendri* SP2 (PV400499.1) and *Trichoderma atroviride* SP1 (PV400486.1) achieved approximately 75% degradation of selected PAHs. Furthermore, HPLC analysis confirmed that these isolates effectively degraded all tested PAHs, with degradation rates of approximately 70%. *F. liriodendri* was the most efficient degrader, achieving degradation rates of 68.50 ± 2.34% for naphthalene, 65.26 ± 1.21% for phenanthrene, 69.21 ± 1.45% for pyrene, and 66.89 ± 1.98% for anthracene. The PAH degradation byproducts of the selected fungal isolates were non-toxic to *Artemia salina*, confirming their environmental safety. These results highlight the bioremediation potential of endophytic fungi isolated from *A. ampeloprasum* and *B. oleracea* var. *capitata* in PAH-contaminated environments.

## 1. Introduction

Air pollution is a significant global environmental issue in the modern world, stemming from the release of contaminants into the atmosphere, thereby compromising the quality of the atmosphere by altering its natural characteristics. The immense growth of urbanization and industrialization causes this rapid air pollution [1]. Sri Lanka has experienced a gradual increase in air pollution over the past few decades. This is due to the widespread development of cities, along with the specific industries and overdevelopment of transportation systems across the country [2].

Polycyclic aromatic hydrocarbons (PAHs) are major hazardous air pollutants, primarily emitted by vehicular traffic [3]. Due to their high toxicity and carcinogenicity, PAHs pose serious health risks to all living organisms [4]. Naphthalene, phenanthrene, anthracene, fluoranthene, pyrene, benzo[a]pyrene, chrysene, and benzo[b]fluoranthene are the most prevalent PAHs that tend to accumulate in the environment [5]. These PAHs can be deposited through either dry or wet deposition on various surfaces, including soil, vegetation, and water resources. The dry deposition of PAHs is commonly detected on the phyllosphere of plants located along roadsides, particularly in areas with heavy traffic congestion [6]. PAH compounds present in the vapour phase of the atmosphere can enter leaves either through stomata uptake or by diffusing through wax layers and membranes. Wet deposition serves as the atmospheric removal process for PAHs, with precipitation efficiently scavenging both particulate and gaseous PAHs from the atmosphere [7]. In Sri Lanka, especially in upcountry regions, many plantations, including those cultivating vegetables, are situated along roadsides. This may cause a significant threat due to the dry deposition of PAHs on the phyllosphere of green leafy vegetables [6].

According to the literature, several researchers have found that many phyllosphere and endophytic microorganisms can degrade and utilize PAHs as their sole carbon source [8,9]. This process, known as “natural bioremediation”, can serve as a technology for cleaning up contaminated environments using such potential microorganisms [10]. Although various microorganisms are involved in bioremediation, fungi are considered the most effective due to the presence of enzymes that are responsible for PAH degradation [11]. Primarily, they achieve this through the production and secretion of different types of ligninolytic enzymes, such as laccase, lignin peroxidase, and manganese peroxidase [12]. Using these enzymes, PAHs can be biotransformed into non-toxic inorganic compounds through mineralization [13].

In Sri Lanka, several investigations have been carried out to explore the bioremediation potential of endophytic and phyllosphere-inhabiting fungi [6,8,14]. For example, in a recent study, the fungus *Phyllosticta capitalensis* was identified as the most effective degrader of pyrene and anthracene, capable of remediating PAHs deposited on the phyllosphere of tea leaves in Sri Lanka [14]. However, apart from a few reported studies mentioned above, there is no substantial evidence regarding the ability of endophytes in green leafy vegetables to degrade deposited PAHs. Upcountry vegetable cultivation is widely practiced in the central highlands of Sri Lanka, particularly in the districts of Nuwara Eliya, Badulla, Kandy, and Matale, where vegetable farming represents a major agricultural activity [15]. Among these districts, Nuwara Eliya makes a significant contribution to the country’s vegetable production [15,16]. Commonly cultivated leafy vegetables in this region include *Allium ampeloprasum* (leeks) and *Brassica oleracea* var. *capitata* (cabbage), which are among the most abundant vegetable crops grown in the upcountry area [16].

*Allium ampeloprasum* (family Amaryllidaceae) grows well in cool climates and prefers well-drained, fertile soils. It is highly adaptable to a range of soil types and environmental conditions; however, it requires adequate and consistent moisture for optimal growth [17]. Similarly, *Brassica oleracea* var. *capitata* (family Brassicaceae) is a cool-season crop commonly grown in spring and autumn in temperate regions. It prefers well-drained, fertile soil with a pH of 6.0–7.5 and requires adequate sunlight for proper growth and development [18]. The cool climate and favorable soil conditions of the central highlands support year-round vegetable cultivation [16]. However, due to limited land availability and increasing demand for vegetables, farmers often cultivate crops along roadsides as an alternative practice. Such cultivation near roadways may expose crops to vehicular emissions, leading to PAH contamination.

Therefore, the present research aimed to isolate and identify endophytic fungal strains inhabiting two of the most consumed leafy vegetables, *A. ampeloprasum* and *B. oleracea* var. *capitata*, in urbanized upcountry areas of Sri Lanka, and to investigate their ability to degrade four PAHs: Naphthalene, Phenanthrene, Anthracene, and Pyrene.

## 2. Materials and Methods

### 2.1. Study Sites

**Figure 1:**
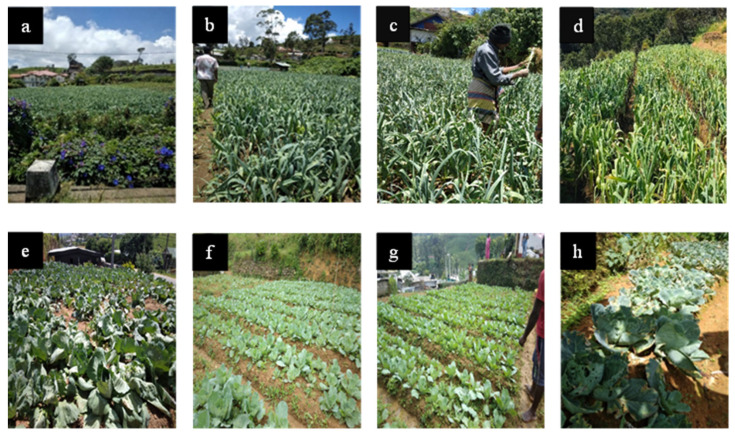
Study sites: *Allium ampeloprasum* bed in (**a**) Nuwara Eliya town; (**b**) Nanu Oya; (**c**) St Clair’s; (**d**) Meepilimana, and *Brassica oleracea* var. *capitata* bed in (**e**) Nuwara Eliya town; (**f**) Nanu Oya; (**g**) St Clair’s; (**h**) Meepilimana.

This study was conducted in the upcountry region of Sri Lanka, with a specific focus on Nuwara Eliya. Three urbanized study sites were selected within Nuwara Eliya city: Nuwara Eliya town, Nanu Oya, and St. Clair’s. Control sites were selected in Meepilimana, located approximately 6 km from Nuwara Eliya town, where *Allium ampeloprasum* and *Brassica oleracea* var. *capitata* were cultivated (Figure 1, Supplementary Figure S1, Supplementary Table S1).

### 2.2. Sample Collection

Three vegetable beds of *A. ampeloprasum* and *B. oleracea* var. *capitata* were randomly selected from each study site. In May 2024, two independent above-ground leafy samples were collected from each bed using a clean knife, resulting in six biological replicates per site. This hierarchical sampling design ensured that samples from different beds were treated as independent replicates. The samples from each site were placed separately in clean, labelled polythene bags, transported in an icebox to the Department of Plant and Molecular Biology, University of Kelaniya, and stored at 4 °C until further analysis. The mean values for subsequent experiments were calculated through statistical analysis.

### 2.3. High Performance Liquid Chromatography (HPLC) Analysis of the Deposited PAH Pollutants on the Phyllosphere of Leaf Samples

Ten grams of freshly collected *A. ampeloprasum* and *B. oleracea* var. *capitata* leaves from each sampling site were separately immersed in 100 mL of a hexane:acetone mixture (1:1, *v*/*v*) in 250 mL Erlenmeyer flasks. The mixtures were shaken for 1 h at 180 rpm using an orbital shaker (MaxQ 4000, Thermo Fisher Scientific, Waltham, MA, USA). The leaf washings were then filtered using a filtration apparatus, and the filtrates were transferred into sterile 250 mL beakers and evaporated at 55 °C. The resulting residues were re-dissolved in 1 mL of acetonitrile (HPLC grade, Merck KGaA, Darmstadt, Germany) and filtered through 0.22 µm nylon syringe filters. The filtrates were collected in 2 mL glass vials for further analysis.

The concentrations of naphthalene, phenanthrene, anthracene, and pyrene in each sample were subsequently determined using an Agilent 1100 Series HPLC system (Agilent, Santa Clara, CA, USA) equipped with an Agilent 1200 diode array detector and a C18 column (4.6 mm × 100 mm) (Agilent, Santa Clara, CA, USA). An acetonitrile:deionized water mixture (90:10, *v*/*v*) was used as the mobile phase at a flow rate of 1.0 mL min^−1^. The HPLC analysis was performed with a 20 µL injection volume, a column temperature of 22 °C, and the detector set at 254 nm for compound detection (bandwidth 4 nm). Recovery for all four PAH standards exceeded 95%, indicating good method accuracy. Peak purity was verified by comparison with standards based on retention times (naphthalene 1.520 min, anthracene 1.910 min, phenanthrene 1.986 min, and pyrene 2.409 min), peak areas, and the presence of clean, narrow peaks without noise [8,19]. Calibration curves were constructed using standards of naphthalene, phenanthrene, anthracene, and pyrene (HPLC grade, Sigma-Aldrich, St. Louis, MO, USA) at known concentrations (Supplementary Figures S2.1–S2.4).

### 2.4. Isolation and Identification of Leaf Endophytic Fungi

A surface sterilization procedure was carried out separately for mature leaf samples of *A. ampeloprasum* and *B. oleracea* var. *capitata* collected from each study site before the isolation of fungal endophytes, using a modified method described by Sari et al. [20]. Each leaf sample was cut into small pieces (1 cm × 1 cm) using a sterilized scalpel and washed under running tap water for 10 min. The leaf pieces were then immersed in 70% ethanol for 30 s, followed by 0.025% (*w*/*v*) aqueous sodium hypochlorite for 10 s, and rinsed again in 70% ethanol for 30 s. Thereafter, the samples were washed with sterile distilled water through three serial rinses of 3 min each.

The surface-sterilized leaf pieces were dried under aseptic conditions, and the edges were trimmed. The leaves were then aseptically cut into 5 mm × 5 mm segments using a sterilized scalpel. Twenty randomly selected 5 mm × 5 mm leaf segments from each vegetable species at each of the four study sites were separately placed on sterile glass Petri dishes containing Potato Dextrose Agar (PDA) (Oxoid, Basingstoke, UK), with four segments per dish. The plates were incubated at room temperature (28 °C) for 3–7 days.

Each fungal species emerging from the plated leaf segments was subcultured onto PDA to obtain pure cultures. Once pure cultures of leaf endophytic fungi were established, they were subjected to morphological examination. The sticky tape method described by Flegel [21] was used to prepare specimens for microscopic observation. Prepared slides were examined under high-power magnification (10 × 40) using a light microscope (Nikon Eclipse E100, Tokyo, Japan). Based on colony morphology and microscopic characteristics of fungal hyphae, spores, and sporulating structures, the fungi were identified to the genus level using the identification keys provided in the Compendium of Soil Fungi [22].

### 2.5. Calculation of the Frequency of Occurrence of Endophytic Fungi

The percentage frequency of occurrence of each endophytic fungal strain was calculated using Equation (1), described by Dharmasiri et al. [19].

(1) $$\mathrm{Frequency}\;\mathrm{of}\;\mathrm{occurrence}\;\%=\;\frac{\mathrm{Number}\;\mathrm{of}\;\mathrm{pieces}\;\mathrm{colonized}\;\mathrm{by}\;\mathrm{the}\;\mathrm{fungus}}{\mathrm{Total}\;\mathrm{number}\;\mathrm{of}\;\mathrm{pieces}\;\mathrm{plated}}\;\times \;100$$

### 2.6. Screening of PAH Degradation Ability of the Endophytic Fungal Strains Through Plate Assay

The ability of each fungal isolate to utilize and degrade selected pure PAH substrates was examined qualitatively following the method described by Kannangara et al. [6]. Each fungal strain was subjected to a 5-day starvation period before further analysis. Selected fungal strains were inoculated onto sterile Petri dishes containing Bacto Bushnell–Haas (BBH) growth medium (HiMedia, Mumbai, India) and incubated for 5 days at room temperature (28 °C).

To assess the capacity of each fungal strain to utilize four different PAHs: naphthalene, phenanthrene, anthracene, and pyrene, four separate BBH plates were prepared for each strain. The BBH plates were individually supplemented with 100 ppm of naphthalene, phenanthrene, anthracene, or pyrene as the sole carbon source. Preliminary experiments using PAH concentrations of 25–150 ppm were conducted to assess fungal tolerance and degradation efficiency. Among the tested concentrations, 100 ppm showed the highest degradation potential without inhibitory effects on fungal growth; therefore, it was selected for subsequent experiments. Four mycelial discs (5 mm × 5 mm) were obtained from each fungal strain following the 5-day starvation period in BBH medium. Control plates for each PAH, without fungal inoculation, were prepared to ensure the accuracy of the results.

The inoculated plates were incubated at room temperature (28 °C) for 3 days. The diameter of each fungal colony was measured after the incubation period. The average colony diameter was calculated as the mean of the four measurements. Six replicates were performed for each fungal strain.

### 2.7. Screening of PAH Degradation Ability of the Endophytic Fungal Strains Through Spectrophotometric Assessment

To evaluate the PAH degradation potential of the fungal isolates, a spectrophotometric assay was conducted following the method described by Undugoda et al. [14], with minor modifications. A total of 50 mL of prepared BBH broth was transferred into each 250 mL Erlenmeyer flask and autoclaved at 120 °C for 15 min. Subsequently, 0.5 mL of 100 ppm naphthalene, phenanthrene, anthracene, and pyrene standards were separately added to their respective autoclaved flasks under aseptic conditions. Two agar plugs (1 cm^2^ each) of each fungal strain from the BBH plates, which had undergone a 5-day starvation period, were aseptically transferred into the respective flasks containing PAHs. Methylene blue (2% *v*/*v*) and Tween 80 (0.1% *v*/*v*) were added to facilitate PAH dispersion and to serve as a redox indicator. Control flasks for each PAH were prepared without fungal inoculation. The flasks were incubated at room temperature (28 °C) with constant shaking at 180 rpm for 7 days. Six replicates were performed for each fungal strain. After incubation, each culture was filtered using a filtration apparatus to separate biomass and other residues, followed by centrifugation at 8000 rpm for 15 min (Model BKC-TL4MII, Biobase, Jinan, China). The absorbance of the supernatant was measured using a UV—Visible spectrophotometer (Thermo Scientific Multiskan GO 1510, Waltham, MA, USA) at the wavelength of 609 nm. The reduction in absorbance of methylene blue was used as an indirect indicator of microbial metabolic activity associated with PAH degradation rather than a direct measurement of PAH concentration. The percentage biodegradation was calculated using Equation (2).

(2) $$\mathrm{Biodegradation}\;\%=\;[1\;-\;\frac{\mathrm{The}\;\mathrm{absorbance}\;\mathrm{of}\;\mathrm{the}\;\mathrm{treated}\;\mathrm{sample}}{\mathrm{The}\;\mathrm{absorbance}\;\mathrm{of}\;\mathrm{control}}]\;\times \;100$$

### 2.8. High Performance Liquid Chromatography (HPLC) Assessment of PAH Degradation Ability of the Endophytic Fungal Strains

HPLC analysis was performed to evaluate the ability of the selected fungal isolates to degrade pyrene, naphthalene, phenanthrene, and anthracene. For each assay, 1 mL of a 5-day-starved fungal culture was inoculated into 50 mL of BBH broth supplemented with 100 ppm of the respective PAH. The cultures were incubated at 28 °C for seven days. After incubation, the mixtures were passed through 0.22 μm nylon syringe filters, and the remaining PAHs were extracted with an acetone–hexane mixture (1:1). The solvent was subsequently removed by rotary evaporation, and the resulting residue was reconstituted in 1 mL of HPLC-grade acetonitrile [8,19]. Samples were then analyzed using an HPLC system equipped with a diode array detector, following the procedure outlined in Section 2.3. PAH degradation was expressed as the percentage reduction relative to the initial concentration of 100 ppm.

### 2.9. Toxicity Assay of Fungal Degradation By-Products Using Artemia Salina

The toxicity of metabolites produced during PAH degradation by fungi was evaluated using an *Artemia salina* (brine shrimp) bioassay. Approximately 1 g of *A. salina* cysts (brine shrimp eggs) was incubated in natural freshwater supplemented with 30 g L^−1^ NaCl in a separation funnel. The solution was continuously aerated using an air pump and maintained at room temperature under constant illumination for 24 h to facilitate hatching.

After 48 h, the hatched nauplii separated from the eggshells and accumulated near the illuminated side of the separation funnel (close to the light source). The nauplii were then collected for the toxicity assay.

For the experiment, 1 mL of 5-day-starved fungal cultures was inoculated into 50 mL of BBH broth supplemented with 100 ppm of the respective PAH and incubated for seven days. For the positive control, 100 ppm of the respective PAH was prepared in BBH broth without fungal culture. For the negative control, BBH broth without PAH was used. After incubation, the PAH-containing fungal culture broth was centrifuged at 10,000 rpm for 3 min to remove fungal biomass, and the resulting supernatant was transferred into sterile glass plates. Ten *Artemia* nauplii were introduced into each plate containing the culture filtrate using a micropipette. The number of surviving nauplii was recorded at hourly intervals for a total period of 6 h [19]. All experiments were performed in triplicate to ensure reproducibility.

### 2.10. Molecular Identification of Fungal Strains

One gram of fungal biomass was carefully scraped from the colony and transferred into a sterilized grinder. It was ground with 600 µL of nucleic lysis solution, then incubated at 65 °C in a water bath for 15 min. Following lysis, 3 µL of RNase solution was added, and the sample was incubated at 37 °C for an additional 15 min. The mixture was then allowed to cool to room temperature (28 °C) for 5 min before 200 µL of protein precipitation solution was added. After thorough vortexing for 1 min, the sample was centrifuged at 13,000 rpm for 3 min.

The resulting supernatant was transferred to a clean tube containing 600 µL of isopropanol, gently mixed, and centrifuged again at 13,000 rpm for 1 min to precipitate the DNA. The supernatant was discarded, and the DNA pellet was washed with ethanol, then air-dried at room temperature. Finally, the DNA pellet was rehydrated in 100 µL of DNA rehydration solution and incubated at 65 °C for 1 h. For PCR amplification, a 25 µL reaction mixture was prepared, containing 2 µL of extracted DNA, 1 µL each of ITS1 (5′-TCCGTAGGTGAACCTGCGG-3′) and ITS4 (5′-TCCTCCGCTTATTGATATGC-3′) primers, 12.5 µL of GoTaq Green Master Mix (M7122, Promega, Madison, WI, USA), and 8.5 µL of nuclease-free water. Amplification was carried out using a Lifeco thermal cycler (Model TC-96/G/H(b)C, BIOER, Hangzhou, China). The PCR products were then separated on 1.5% agarose gels and visualized using a GelDoc image analyzer (Bio-Rad, Hercules, CA, USA). A 2 kb molecular weight ladder (Opti-DNA Marker, Applied Biological Materials Inc., Richmond, BC, Canada) was used as the molecular weight marker.

The resulting PCR products were sent to Macrogen (Korea) for sequencing. The sequences were compared with the standard sequences in the NCBI GenBank database using the BLASTN tool (https://blast.ncbi.nlm.nih.gov/Blast.cgi?PROGRAM=blastn&PAGE_TYPE=BlastSearch&LINK_LOC=blasthome (accessed on 16 April 2026)) to identify the isolated fungal species.

### 2.11. Statistical Analysis

Data obtained from the colorimetric assay, plate assay, and HPLC analysis were expressed as means of six biological replicates. Prior to statistical analysis, data normality was assessed using the Shapiro–Wilk test, and homogeneity of variance was evaluated using Levene’s test. One-way analysis of variance (ANOVA) was performed using Minitab 17 software at a significance level of *p* < 0.05. When significant differences were detected, means were compared using the least significant difference (LSD) test.

For multivariate analysis, principal component analysis (PCA) and heat maps were generated using OriginPro 11 software. Before performing PCA, the data were standardized (z-score scaling) to ensure equal weighting of variables and to avoid bias due to differences in measurement scales.

## 3. Results and Discussion

**Figure 2:**
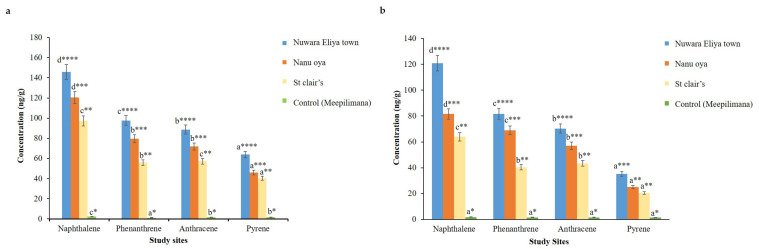
Concentrations of different PAHs in the phyllosphere of (**a**) *Allium ampeloprasum* and (**b**) *Brassica oleracea* var. *capitata* collected from different study sites. Different alphabetical letters indicate significant differences among PAHs within the same study site, and varying numbers of asterisks (*) indicate significant differences for a given PAH among different study sites (*p* < 0.05).

In this study, the amounts of deposited PAHs (naphthalene, phenanthrene, anthracene, and pyrene) in the phyllosphere of *A. ampeloprasum* and *B. oleracea* var. *capitata* grown along roadsides, as well as in a rural area (Meepilimana), were evaluated. In agreement with Undugoda et al. [23], the present study demonstrated significantly higher concentrations of the tested PAHs in the phyllosphere of leafy vegetables collected from roadsides in urbanized areas compared to those from the unurbanized rural area located approximately 6 km from Nuwara Eliya town. Figure 2 illustrates the differences in PAH accumulation across the study sites. For all four PAHs, Nuwara Eliya town exhibited the highest concentrations (*p* < 0.05), followed by Nanu Oya and St. Clair’s, while only trace levels were detected at the control site. Among the four compounds, naphthalene showed the highest accumulation at all sites (*p* < 0.05). Overall, PAH contamination varied among sites, with the highest levels observed in the more polluted locations.

The phyllosphere of *A. ampeloprasum* collected from Nuwara Eliya town showed the significantly highest naphthalene concentration (*p* < 0.05), with a value of 145.92 ng/g. Nanu Oya and St. Clair’s recorded concentrations of 120.42 ng/g and 97.26 ng/g, respectively. Leaf samples collected from the control site exhibited the lowest phyllosphere concentrations of naphthalene, phenanthrene, anthracene, and pyrene, with values of 2.35 ng/g, 1.26 ng/g, 1.64 ng/g, and 1.70 ng/g, respectively. *B. oleracea* var. *capitata* leaves showed a similar pattern of phyllosphere PAH concentrations, with the highest naphthalene, phenanthrene, anthracene, and pyrene levels observed in samples from Nuwara Eliya town, at 120.87 ng/g, 81.74 ng/g, 70.32 ng/g, and 35.20 ng/g, respectively. Compared to *B. oleracea* var. *capitata*, the phyllosphere of *A. ampeloprasum* contained higher PAH accumulation, likely due to its growth habit and physiological characteristics. The higher PAH concentrations observed in *A. ampeloprasum* compared to *B. oleracea* var. *capitata* may be attributed to differences in plant morphology and exposure pathways. In *A. ampeloprasum*, the edible pseudostem develops partially below the soil surface, potentially increasing contact with environmental PAHs and facilitating the accumulation of hydrophobic compounds like PAHs in lipid-rich tissues. In contrast, the above-ground edible parts of *B. oleracea* are mainly exposed to atmospheric deposition, which may result in comparatively lower accumulation. Although soil PAH levels were not analyzed in the present study, the findings suggest that plant structural characteristics and compound partitioning behavior likely influenced the observed differences [24].

**Figure 3:**
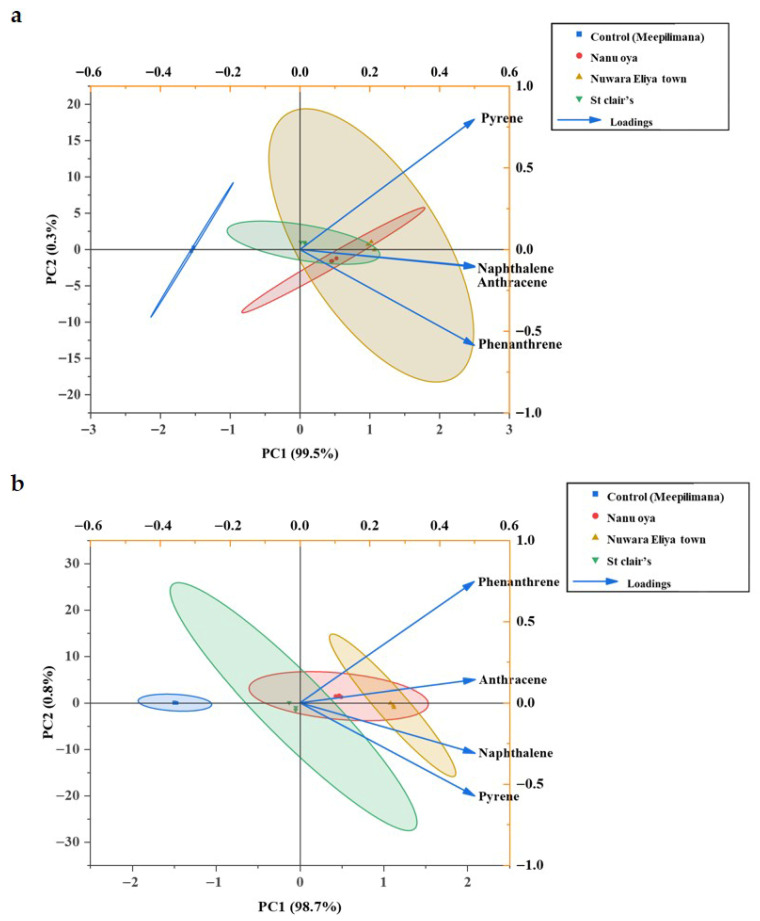
The principal component analysis (PCA) on the distribution of the PAHs pollutants in the phyllosphere of (**a**) *Allium ampeloprasum* and (**b**) *Brassica oleracea* var. *capitata* based on the sampling sites. Different colours in the PCA ellipse represent different sampling sites.

Principal component analysis (PCA) was employed to assess the distribution patterns of four PAHs (naphthalene, phenanthrene, anthracene, and pyrene) across three highly polluted sites and a control site (Figure 3). For *A. ampeloprasum* (Figure 3a), the PCA biplot explained 99.8% of the total variance, with PC1 and PC2 accounting for 99.5% and 0.3%, respectively. PC1 clearly separated the polluted sites: Nuwara Eliya Town, Nanu Oya, and St. Clair’s, from the control site (Meepilimana), indicating substantially higher concentrations of all four PAHs in samples from the polluted areas. PC2 showed positive loadings for pyrene, while naphthalene, phenanthrene, and anthracene exhibited negative loadings. Nuwara Eliya Town displayed the highest PAH concentrations, likely due to intense urbanization and vehicular emissions. Similarly, for *B. oleracea* var. *capitata* (Figure 3b), the PCA explained 99.5% of the total variance, with PC1 contributing 98.7% and PC2 contributing 0.8%. PC1 highlighted Nuwara Eliya Town and Nanu Oya on the positive axis, reflecting elevated PAH levels compared to other sites, while PC2 showed positive loadings for phenanthrene and anthracene and negative loadings for naphthalene and pyrene. Across both leafy vegetables, samples from Nuwara Eliya Town consistently exhibited the highest PAH contamination, attributable to greater urbanization and vehicular traffic compared to Nanu Oya and St. Clair’s, whereas the control site remained distinctly separated with minimal PAH pollution.

After assessing pollution distribution among urban areas, the frequency of occurrence of leaf endophytic fungi was evaluated (Table 1 and Table 2). Sixteen fungal endophytes were isolated from *A. ampeloprasum* leaves collected from the upcountry area (Table 1) and belonged to 11 genera, including *Trichoderma*, *Fusarium*, *Aspergillus*, *Cochliobolus*, *Pestalotiopsis*, *Humicola*, and *Acremonium*, along with three sterile morphotypes categorized as white sterile (unidentified genus 1), gray sterile (unidentified genus 2), and brown sterile (unidentified genus 3) as mentioned in the preliminary study results presented by Amarasekara et al. [25]. In *B. oleracea* var. *capitata*, thirteen endophytic fungi were isolated. Most of the fungal endophytes recovered from *B. oleracea* var. *capitata* were similar to those isolated from *A. ampeloprasum*. *Penicillium* was the only additional genus recorded from *B. oleracea* var. *capitata* in the upcountry area compared to *A. ampeloprasum* (Table 2). The highest frequency of occurrence was observed for endophytic fungi isolated from the highly PAH-polluted site, Nuwara Eliya town, compared to the less polluted sites and the control site, indicating that the most frequently occurring fungal species were associated with highly polluted areas.

Overall, a relatively small number of fungal endophytes were isolated. Among them, *A. ampeloprasum* exhibited higher diversity and frequency of occurrence compared to *B. oleracea* var. *capitata*. This difference may be partly due to the higher accumulation of PAHs in the phyllosphere of *A. ampeloprasum*, which could favour the presence of PAH-tolerant or PAH-utilizing fungal endophytes. In addition, the higher isolation of endophytic fungi from *A. ampeloprasum* can be attributed to intrinsic differences in plant chemistry and tissue structure. *A. ampeloprasum* contains sulfur-based compounds that tend to select for tolerant endophytic fungi rather than exclude them, and its soft, moisture-rich tissues provide a favourable environment for fungal establishment [26,27]. In contrast, *B. oleracea* produces glucosinolates with strong antifungal activity and has compact leaf tissues, both of which limit fungal entry and persistence, resulting in lower endophytic fungal diversity [28,29].

Upcountry vegetable farming is an intensive production system that relies heavily on pesticides and fertilizers, largely due to the short growth cycles of crops and humid conditions that strongly favor the rapid spread of pests and diseases. It has been reported that approximately 40% of farmers frequently apply pesticides, including fungicides, as a precautionary measure prior to the appearance of any pest or fungal disease symptoms. Furthermore, pesticide overdosing has been reported in 38% and 41% of farmers in the Badulla and Nuwara Eliya districts, respectively [30]. The frequent use of pesticides and fungicides may inhibit not only fungal pathogens but also beneficial and non-pathogenic fungal endophytes in crop plants, which could explain the low abundance of leaf endophytes observed in the present study.

Numerous studies have reported that the composition of fungal endophyte consortia varies according to substrate type [31,32]. In recent research conducted by Undugoda et al. [14], several fungal endophytes belonging to the genera *Colletotrichum*, *Daldinia*, *Phyllosticta* and *Pseudopestalotiopsis* were isolated from tea leaves in the upcountry region of Sri Lanka. These findings explain why a completely different consortium of fungal populations is present in tea leaves compared to the leaves of the leafy vegetables used in the present study, despite similar geographical and climatic conditions. Leaf endophytes are largely host-specific, meaning that many bacteria and fungi typically associate with plant species based on host chemical profiles [33].

**Figure 4:**
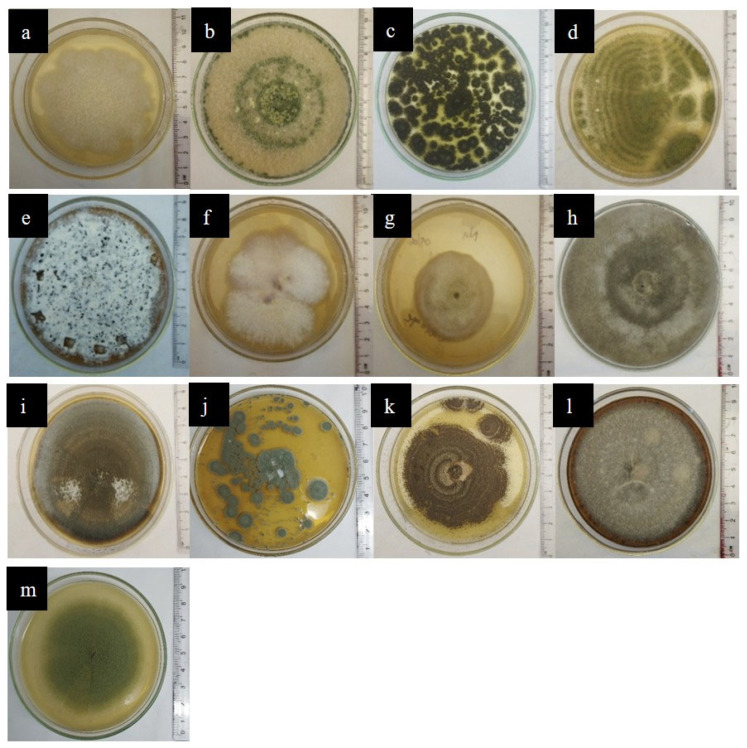
Fungal cultures of (**a**) *Fusarium liriodendri* SP2 (PV400499.1), (**b**) *Trichoderma atroviride* SP1 (PV400486.1), (**c**) *Aspergillus* sp.1, (**d**) *Aspergillus* sp.2, (**e**) *Pestalotiopsis* sp.1, (**f**) *Humicola* sp.1, (**g**) *Acremonium* sp.1, (**h**) Gray sterile sp.1, (**i**) *Cochliobolus* sp.1, (**j**) *Penicillium* sp.1, (**k**) *Aspergillus* sp.4, (**l**) Gray sterile sp.2 (**m**) *Trichoderma* sp.2.

Out of twenty-two isolated endophytic fungi, the thirteen most frequently occurring strains from both *A. ampeloprasum* and *B. oleracea* var. *capitata* were selected for the plate assay and spectrophotometric analysis. The morphological characteristics of these strains are shown in Figure 4.

The plate assay results (Table 3) revealed significant differences (*p* < 0.05) among the isolates in their ability to utilize the four selected PAHs as the sole carbon source. Among them, *Fusarium liriodendri* SP2 and *Trichoderma atroviride* SP1, which exhibited the significantly highest frequency of occurrence, also showed the highest growth diameters on all PAH-supplemented media (*p* < 0.05). Following these, *Aspergillus* sp. 1, *Aspergillus* sp. 2, *Penicillium* sp. 1, and Gray sterile sp. 1 exhibited high growth diameters, while the remaining fungal isolates showed moderate growth.

**Figure 5:**
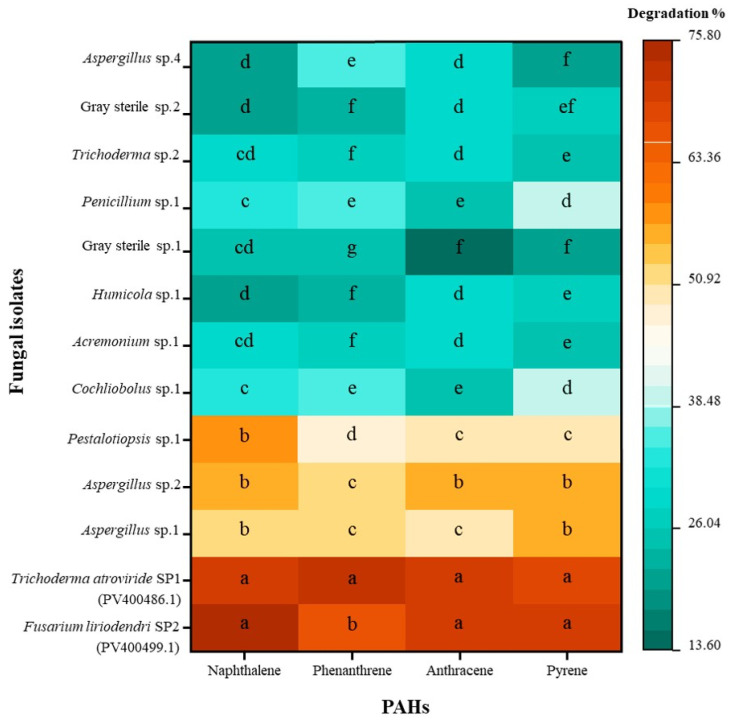
Heatmap of PAHs degradation percentage of fungal strains isolated from *Allium ampeloprasum* and *Brassica oleracea* var. *capitata* in spectrophotometric analysis (*n* = 6). Different alphabetic letters within the same column are considered significantly different at a significance level (*p* < 0.05). Colour variations represent the difference in degradation ability of each fungal strain. For the two identified strains, the GenBank accession numbers are provided in brackets.

The colourimetric method applied in the present investigation demonstrated that most of the isolated fungal endophytes efficiently degraded all four substrates: naphthalene, phenanthrene, anthracene, and pyrene, to varying extents (Figure 5). Among them, *Fusarium liriodendri* SP2, *Trichoderma atroviride* SP1, *Aspergillus* sp. 1, and *Aspergillus* sp. 2 exhibited the highest degradation abilities, each achieving more than 50% degradation of all four PAHs. Notably, *F. liriodendri* SP2 and *T. atroviride* SP1 showed significantly higher degradation efficiencies (*p* < 0.05), exceeding 70% for all PAHs. *F. liriodendri* SP2 exhibited significantly higher degradation percentages (*p* < 0.05) for naphthalene, anthracene, and pyrene compared with phenanthrene, whereas *T. atroviride* SP1 showed consistently high degradation with no significant differences (*p* > 0.05) among the four PAHs. Other fungal strains, including *Cochliobolus* sp. 1, *Acremonium* sp. 1, *Humicola* sp. 1, and Gray sterile sp. 1, demonstrated significantly lower degradation efficiencies. As identified in previous studies, the potential degradation of PAHs by fungi may be attributed to two main mechanisms: the involvement of the cytochrome P450 system [34] and the action of soluble extracellular enzymes associated with lignin degradation, including lignin peroxidase and manganese peroxidase [35].

**Figure 6:**
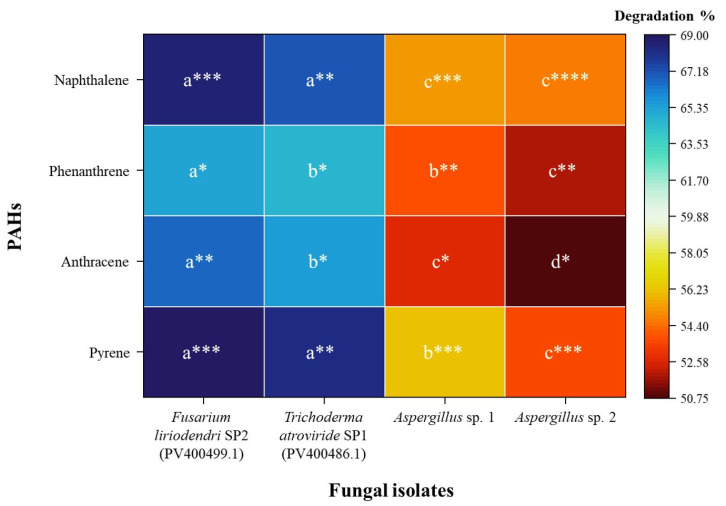
Heatmap of PAH degradation percentages by fungal strains isolated from *Allium ampeloprasum* and *Brassica oleracea* var. *capitata*, determined by HPLC analysis (*n* = 3). Different letters indicate significant differences among fungal strains for the same PAH, and varying numbers of asterisks (*) indicate significant differences among PAHs for the same fungal strain (*p* < 0.05). Colour intensity represents the relative degradation ability of each fungal strain. For the two identified strains, the GenBank accession numbers are provided in brackets.

After screening using plate and spectrophotometric assays, four fungal isolates with high degradation potential were selected: *F. liriodendri* SP2, *T. atroviride* SP1, *Aspergillus* sp. 1, and *Aspergillus* sp. 2. Their PAH-degrading abilities were confirmed through HPLC analysis. As shown in Figure 6, *F. liriodendri* SP2 demonstrated the highest degradation efficiency for naphthalene (68.50 ± 2.34%) and pyrene (69.21 ± 1.45%), indicating a strong capacity for selective PAH degradation. Degradation of phenanthrene (65.26 ± 1.21%) and anthracene (66.89 ± 1.98%) by this isolate was slightly lower but still significant, suggesting substrate-specific variation in metabolic activity. In contrast, *T. atroviride* SP1 showed slightly lower degradation efficiencies than *F. liriodendri* SP2 but maintained consistently high degradation across all four PAHs. *Aspergillus* sp. 1 and *Aspergillus* sp. 2 showed moderate degradation of all tested PAHs, with slightly higher activity toward naphthalene and pyrene than toward anthracene and phenanthrene. Overall, these results indicate that *F. liriodendri* SP2 and *T. atroviride* SP1 are the most promising candidates for targeted PAH bioremediation, exhibiting high efficiency and broad-spectrum activity, whereas the *Aspergillus* isolates demonstrate moderate to low PAH removal potential.

Notably, the two fungal strains with the highest PAH-degrading potential, *F. liriodendri* SP2 and *T. atroviride* SP1, were both isolated solely from *A. ampeloprasum*, which showed the highest PAH concentrations in leaf wash samples (Figure 2, Table 1). This indicates that these strains may have greater tolerance to PAHs, allowing for higher degradation efficiencies. Furthermore, the results of this study confirm that fungi with higher PAH degradation ability developed comparatively larger colony diameters on PAH-supplemented media, whereas poor degraders exhibited significantly smaller colony diameters (Table 3), indicating a positive correlation between PAH degradation capacity and colony diameter.

According to the results shown in Table 4, after 6 h of exposure, all four fungal treatments showed complete survival of the nauplii (10/10) for phenanthrene, anthracene, naphthalene, and pyrene, indicating that the metabolites generated during PAH degradation were not toxic. In contrast, the positive control containing untreated 100 ppm PAH showed reduced nauplii survival, confirming the toxic effect of the parent compounds, while the negative control showed complete survival. These results suggest that the byproducts formed after PAH degradation by the four selected fungal isolates were non-toxic to *Artemia salina*. Therefore, these fungal isolates demonstrate potential for environmentally safe PAH phylloremediation applications.

From the above analyses, the two selected fungal isolates, *Fusarium liriodendri* SP2 and *Trichoderma atroviride* SP1, were further confirmed at the molecular level using the GenBank BLASTN tool by comparison with related fungal species isolated from plant sources (Table 5).

To support the present findings, previous studies have reported that *Fusarium* species are capable of degrading PAHs, potentially due to their ability to produce a broad range of oxidative and detoxifying enzymes, including cytochrome P450s, laccases, and peroxidases [36]. They can tolerate and adapt to hydrocarbon-polluted environments by storing and transforming PAHs inside their cells, allowing them to survive and function even under heavy contamination [37]. In particular, *F. solani* isolate MM1 has identified as a model strain for its ability to degrade PAHs such as phenanthrene and pyrene by co-metabolizing with glucose [38]. Additionally, *Fusarium* performs well both in pure cultures and in mixed fungal–bacterial consortia, where complementary metabolic pathways often enhance overall PAH breakdown [39]. Because they are frequently isolated from contaminated soils and ecologically adapted to polluted environments, further supporting their suitability for environmental bioremediation [10]. Similarly, several *Trichoderma* species are known to degrade a wide range of PAHs, including both low and high molecular weight compounds such as naphthalene, phenanthrene, chrysene, pyrene, and benzo[a]pyrene [40,41]. Species reported to possess PAH-degrading ability include *T. hamatum*, *T. harzianum*, *T. reesei*, *T. koningii*, *T. viride*, *T. virens*, and *T. asperellum*. These *Trichoderma* species have been reported to possess enzyme systems such as multicopper laccases, peroxidases, and dioxygenases involved in the efficient degradation of aromatic pollutants [40,41]. Therefore, in this study, these two fungal genera outperformed the other strains.

According to previous findings by Dharmasiri et al. [8], *F. solani* isolate P11M-46 and *T. harzianum* isolate P4M-16, isolated from urban ornamental plants in Sri Lanka, were reported as the most efficient PAH degraders. This observation aligns with the findings of the present study, in which *Trichoderma* sp. and *Fusarium* sp. were the most effective PAH degraders. In contrast, the study by Kannangara et al. [6] highlighted *Penicillium* as the most effective genus, capable of degrading both naphthalene and phenanthrene by more than 85%. Similarly, Undugoda et al. [14] reported that *Penicillium oxalicum* isolated from tea leaves achieved the highest degradation of phenanthrene and naphthalene, at 80% and 96%, respectively. In comparison, the *Penicillium* species isolated in the present study showed relatively low PAH degradation, less than 30%. However, the fungal endophytes isolated in this study might have tolerance to higher concentrations of frequently applied pesticides and fungicides, as Nuweraliya farmers commonly use intensive fungicide treatments for these leafy vegetables. Supporting this notion, Escudero-Leyva et al. [42] reported that *Trichoderma* spp. isolated from living leaf tissues of wild Rubiaceae plants were tolerant to fungicides and efficient in their biological removal.

Based on the findings of the present study, *F. liriodendri* SP2 and *T. atroviride* SP1 can be considered the most effective fungal endophytic strains to degrade naphthalene, phenanthrene, pyrene, and anthracene. This emphasizes *F. liriodendri* SP2 and *T. atroviride* SP1 as the most promising and effective PAH degraders for phylloremediation processes in sustainable agricultural practices in the future. Moreover, the present study opens avenues for further research involving additional PAH compounds and field trials under natural environmental conditions, including variations in soil characteristics and climatic factors. PAH degradation in natural environments is often mediated by complex microbial communities where fungi and bacteria interact synergistically to enhance pollutant breakdown [14,19]. In this study, pure fungal cultures were used to evaluate the intrinsic PAH degradation potential of the isolates under controlled laboratory conditions, which may not fully reflect natural ecosystem interactions. Therefore, future studies should focus on developing fungal–bacterial consortia by combining the selected fungal isolates with efficient PAH-degrading bacterial strains after assessing their compatibility to enhance biodegradation efficiency.

## 4. Conclusions

Vegetable samples collected from roadside urban sites in the Nuwara Eliya district showed significantly higher concentrations of PAHs compared to the control site, indicating the influence of environmental pollution. Naphthalene, phenanthrene, anthracene, and pyrene were detected at elevated levels in the phyllosphere of the selected vegetables. The results further demonstrated that endophytic fungi isolated from the above-ground parts of *Allium ampeloprasum* and *Brassica oleracea* var. *capitata* were capable of degrading these PAHs under laboratory conditions. In particular, *Fusarium liriodendri* SP2 and *Trichoderma atroviride* SP1 showed the highest degradation efficiency, achieving approximately 70% reduction of the tested compounds. These findings indicate that selected endophytic fungi may have potential applications in the bioremediation of PAH-contaminated phyllosphere environments.

## Figures and Tables

**Table 1:** Percentage frequency of occurrence of endophytic fungi isolated from *A. ampeloprasum*.

Endophytic Fungi	Frequency of Occurrence (%)			
	Nuwara Eliya Town	Nanu Oya	St Clair’s	Control (Meepilimana)
*Fusarium liriodendri* SP2 (PV400499.1)	92.5 ± 2.00 ^a^	67.0 ± 0.95 ^b^	48.0 ± 0.42 ^b^	17.5 ± 0.35 ^b^
*Aspergillus* sp.1	85.0 ± 0.75 ^b^	61.5 ± 0.46 ^c^	58.0 ± 0.21 ^a^	31.0 ± 0.22 ^a^
*Trichoderma atroviride* SP1 (PV400486.1)	68.5 ± 0.21 ^c^	72.0 ± 0.53 ^a^	_	_
*Aspergillus* sp.2	59.0 ± 0.63 ^d^	_	32.5 ± 0.36 ^d^	12.5 ± 0.25 ^c^
*Pestalotiopsis* sp.1	41.5 ± 0.46 ^e^	38.5 ± 0.20 ^de^	22.0 ± 0.35 ^g^	_
*Aspergillus* sp.3	20.0 ± 0.58 ^h^	21.0 ± 0.18 ^g^	_	_
*Aspergillus* sp.4	25.0 ± 0.10 ^g^	_	_	_
Gray sterile sp.1	22.5 ± 0.25 ^gh^	37.0 ± 0.32 ^e^	42.0 ± 0.48 ^c^	5.0 ± 0.31 ^d^
*Cochliobolus* sp.1	30.5 ± 0.21 ^f^	10.5 ± 0.18 ^i^	30.5 ± 0.35 ^e^	_
White sterile sp.1	_	_	25.5 ± 0.23 ^f^	_
*Humicola* sp.1	_	29.0 ± 0.25 ^f^	7.0 ± 0.10 ^h^	_
*Acremonium* sp.1	30.5 ± 0.21 ^f^	39.0 ± 0.25 ^d^	5.0 ± 0.31 ^i^	_
Brown sterile sp.1	_	_	21.0 ± 0.43 ^g^	_
*Fusarium* sp.1	2.5 ± 0.40 ^i^	_	_	_
*Aspergillus* sp.5	2.5 ± 0.25 ^i^	15.0 ± 0.32 ^h^	_	_
White sterile sp.2	2.0 ± 0.25 ^i^	3.5 ± 0.23 ^j^	1.5 ± 0.20 ^j^	_

Values represent the mean frequency of occurrence of six samples ± standard deviation. _ indicate the absence of the particular isolate. Different alphabetical letters indicate significant differences among fungal strains within the same study site. For the two identified strains, the GenBank accession numbers are provided in brackets.

**Table 2:** Percentage frequency of occurrence of endophytic fungi isolated from *B. oleracea* var. *capitata*.

Endophytic Fungi	Frequency of Occurrence (%)			
	Nuwara Eliya Town	Nanu Oya	Sent Clair’s	Control (Meepilimana)
*Aspergillus* sp.1	75.0 ± 0.54 ^a^	69.0 ± 0.95 ^a^	55.0 ± 0.48 ^b^	_
*Aspergillus* sp.2	65.0 ± 0.76 ^b^	59.0 ± 0.46 ^b^	58.0 ± 0.12 ^a^	21.0 ± 0.22 ^a^
*Trichoderma* sp.2	42.5 ± 0.26 ^c^	48.5 ± 0.32 ^c^	45.0 ± 0.26 ^c^	20.0 ± 0.25 ^a^
*Aspergillus* sp.4	32.0 ± 0.54 ^d^	23.0 ± 0.18 ^d^	_	_
*Aspergillus* sp.3	22.0 ± 0.63 ^e^	_	_	12.5 ± 0.25 ^b^
*Pestalotiopsis* sp.1	13.5 ± 0.46 ^f^	_	11.5 ± 0.36 ^g^	_
Gray sterile sp.2	_	_	42.0 ± 0.48 ^cd^	5.0 ± 0.31 ^c^
Gray sterile sp.3	12.5 ± 0.21 ^f^	9.0 ± 0.45 ^e^	20.5 ± 0.35 ^f^	_
White sterile sp.1	_	_	25.5 ± 0.23 ^e^	_
*Penicillium* sp.1	_	59.0 ± 0.25 ^b^	39.0 ± 0.25 ^d^	_
*Acremonium* sp.2	12.0 ± 0.19 ^f^	9.0 ± 0.25 ^e^	_	_
Brown sterile sp.2	_	_	21.0 ± 0.43 ^f^	_
*Fusarium* sp.1	1.5 ± 0.25 ^g^	_	_	_

Values represent the mean frequency of occurrence of six samples ± standard deviation. _ indicate the absence of the particular isolate. Different alphabetical letters indicate significant differences among fungal strains within the same study site.

**Table 3:** Growth of fungal colonies isolated from *A. ampeloprasum* and *B. oleracea* var. *capitata* in PAH-containing media at three polluted sites.

Endophytic Fungi	Diameter of Fungal Colonies (mm)			
	Naphthalene	Phenanthrene	Anthracene	Pyrene
*Fusarium liriodendri* SP2 (PV400499.1)	37.83 ± 1.32 ^a^	28.22 ± 1.56 ^ab^	36.67 ± 0.34 ^a^	31.04 ± 1.45 ^a^
*Trichoderma atroviride* SP1 (PV400486.1)	30.16 ± 1.72 ^b^	34.46 ± 2.32 ^a^	31.24 ± 1.86 ^b^	30.69 ±1.32 ^a^
*Aspergillus* sp.1	27.57 ± 1.34 ^bc^	26.42 ± 1.78 ^b^	26.36 ± 1.46 ^c^	25.12 ± 0.35 ^b^
*Aspergillus* sp.2	29.66 ± 4.54 ^bc^	24.56 ± 0.98 ^bc^	25.89 ± 0.57 ^c^	25.54 ± 1.75 ^b^
*Pestalotiopsis* sp.1	20.50 ± 1.51 ^d^	18.90 ± 2.45 ^de^	22.45 ± 0.36 ^cd^	20.57 ± 0.32 ^c^
*Cochliobolus* sp.1	19.33 ± 1.03 ^de^	15.18 ± 1.23 ^e^	15.27 ± 1.34 ^e^	18.36 ± 1.37 ^cd^
*Acremonium* sp.1	26.16 ± 0.75 ^c^	25.95 ± 1.24 ^b^	23.78 ± 0.56 ^cd^	20.19 ± 1.38 ^c^
*Humicola* sp.1	17.00 ± 1.26 ^e^	23.32 ± 1.04 ^c^	20.86 ± 1.89 ^d^	21.58 ± 1.29 ^c^
*Aspergillus* sp.4	12.55 ± 2.94 ^c^	11.43 ± 1.86 ^f^	15.39 ± 1.03 ^e^	16.13 ± 1.49 ^d^
*Penicillium* sp.1	26.11 ± 0.75 ^c^	24.80 ± 1.24 ^bc^	21.59 ± 0.56 ^d^	23.89 ± 1.38 ^bc^
*Trichoderma* sp.2	19.16 ± 1.72 ^de^	20.41 ± 2.32 ^d^	21.64 ± 1.86 ^d^	25.68 ± 1.32 ^b^
Gray sterile sp.2	21.18 ± 1.83 ^d^	23.04 ± 1.35 ^c^	23.78 ± 1.32 ^cd^	21.75 ± 1.23 ^c^
Gray sterile sp.1	28.16 ± 1.83 ^bc^	25.06 ± 1.35 ^bc^	25.78 ± 1.32 ^c^	26.89 ± 1.23 ^b^

Values represent the mean of six replicates ± standard deviation and indicated by different alphabetic letters within the same column are considered significantly different at a significance level (*p* < 0.05). For the two identified strains, the GenBank accession numbers are provided in brackets.

**Table 4:** Survival of *Artemia salina* (*n* = 10) in a toxicity assay of fungal cultures treated with PAHs after 6 h of exposure.

Fungal Species	Number of Live *Artemia Salina*			
	Phenanthrene	Anthracene	Naphthalene	Pyrene
*Fusarium liriodendri* SP2 (PV400499.1)	10/10	10/10	10/10	10/10
*Trichoderma atroviride* SP1 (PV400486.1)	10/10	10/10	10/10	10/10
*Aspergillus* sp.1	10/10	10/10	10/10	10/10
*Aspergillus* sp.2	10/10	10/10	10/10	10/10
Positive control (100 ppm PAH)	8/10	8/10	7/10	8/10
Negative control (without PAH)	10/10	10/10	10/10	10/10

For the two identified strains, the GenBank accession numbers are provided in brackets.

**Table 5:** BLASTN results of isolates related to the ITS region.

Isolates		Similar Strain in BLASTN		Query Cover	E-Value	Percentage of Identification
Name	GeneBank Accession No.	Name	GeneBank Accession No.			
*Fusarium liriodendri* SP2	PV400499.1	*Fusarium liriodendri* isolate PaFS19	PP475083.1	100.00%	0.0	100.00%
*Trichoderma atroviride* SP1	PV400486.1	*Trichoderma atroviride* isolate 22L4TL	PX586810.1	100.00%	0.0	99.76%

## Data Availability

The original contributions presented in the study are included in the article; further inquiries can be directed to the corresponding author.

## Supplementary Materials

The following supporting information can be downloaded at: https://www.mdpi.com/article/10.3390/jof12040295/s1, Figure S1: Map coordinates of selected sample collection sites in Nuwara Eliya district (a) control site—Meeplimana, (b) Nuwara Eliya town, (c) Nanu Oya, (d) St Clair’s; Figure S2: 2.1—Naphthalene standard curve, 2.2—Anthracene standard curve, 2.3—Phenanthrene standard curve, 2.4—Pyrene standard curve; Table S1: Environmental conditions and site coordination details of sample collection area.

## References

[1] Rathnayake L.R.S.D., Sakurat G.B., Weerasekara N.A. (2023). Review of Outdoor Air Pollution in Sri Lanka Compared to the South Asian Region. Nat. Environ. Pollut. Technol..

[2] Senarathna M., Priyankara S., Jayaratne R., Weerasooriya R., Morawska L., Bowatte G. (2022). Measuring Traffic-Related Air Pollution Using Smart Sensors in Sri Lanka: Before and During a New Traffic Plan. Geogr. Environ. Sustain..

[3] Singh A., Banerjee T., Latif M.T., Ramanathan S., Suradi H., Othman M., Murari V. (2023). Molecular Distribution, Sources and Potential Health Risks of Fine Particulate-Bound Polycyclic Aromatic Hydrocarbons during High Pollution Episodes in a Subtropical Urban City. Chemosphere.

[4] Barbosa F., Rocha B.A., Souza M.C., Bocato M.Z., Azevedo L.F., Adeyemi J.A., Santana A., Campiglia A.D. (2023). Polycyclic Aromatic Hydrocarbons (PAHs): Updated Aspects of Their Determination, Kinetics in the Human Body, and Toxicity. J. Toxicol. Environ. Health B.

[5] Rengarajan T., Rajendran P., Nandakumar N., Lokeshkumar B., Rajendran P., Nishigaki I. (2015). Exposure to Polycyclic Aromatic Hydrocarbons with Special Focus on Cancer. Asian Pac. J. Trop. Biomed..

[6] Kannangara S., Undugoda L., Rajapaksha N., Abeywickrama K. (2016). Depolymerizing Activities of Aromatic Hydrocarbon-Degrading Phyllosphere Fungi in Sri Lanka. J. Bioremediat. Biodegrad..

[7] Gong P., Wang X. (2021). Forest Fires Enhance the Emission and Transport of Persistent Organic Pollutants and Polycyclic Aromatic Hydrocarbons from the Central Himalaya to the Tibetan Plateau. Environ. Sci. Technol. Lett..

[8] Strobel G., Booth E., Schaible G., Mends M.T., Sears J., Geary B. (2013). The Paleobiosphere: A Novel Device for the In Vivo Testing of Hydrocarbon Producing–Utilizing Microorganisms. Biotechnol. Lett..

[9] Dharmasiri R.B.N., Undugoda L.J.S., Nilmini A.H.L., Pathmalal M.M., Nugara N.N.R.N., Udayanga D., Kannangara S. (2023). Depolymerization of Polyaromatic Hydrocarbons by *Penicillium* spp. Inhabiting the Phyllosphere of Urban Ornamental Plants. Environ. Qual. Manag..

[10] Fallahi M., Sarempour M., Mirzadi Gohari A. (2023). Potential Biodegradation of Polycyclic Aromatic Hydrocarbons (PAHs) and Petroleum Hydrocarbons by Indigenous Fungi Recovered from Crude Oil-Contaminated Soil in Iran. Sci. Rep..

[11] Vaksmaa A., Guerrero-Cruz S., Ghosh P., Zeghal E., Hernando-Morales V., Niemann H. (2023). Role of Fungi in Bioremediation of Emerging Pollutants. Front. Mar. Sci..

[12] Suryadi H., Judono J.J., Putri M.R., Eclessia A.D., Ulhaq J.M., Agustina D.N., Sumiati T. (2022). Biodelignification of Lignocellulose Using Ligninolytic Enzymes from White-Rot Fungi. Heliyon.

[13] Haritash A.K., Kaushik C.P. (2009). Biodegradation Aspects of Polycyclic Aromatic Hydrocarbons (PAHs): A Review. J. Hazard. Mater..

[14] Undugoda L., Thambugala K., Kannangara S., Munasinghe J., Premarathna N., Dharmasiri N. (2025). Phylloremediation of Pyrene and Anthracene by Endophytic Fungi Inhabiting Tea Leaves (*Camellia sinensis* (L.) Kuntze) in Sri Lanka. N. Z. J. Bot..

[15] Dharmathilake N.R.D.S., Rosairo H.S.R., Ayoni V.D.N., Herath R.M. (2019). Implications of post-harvest losses and acreage response of selected upcountry vegetables from Nuwara Eliya District in Sri Lanka on sustained food security. J. Agric. Sci. Sri Lanka.

[16] Nuskiya F. (2019). Up-country vegetable production and marketing: Challenges and opportunities. SEUSL J. Mark..

[17] Čepulienė V., Juškevičienė D., Viškelis J., Morkeliūnė A., Karklelienė R. (2024). Biological diversity and nutritional importance of *Allium* perennial vegetable species. Sustainability.

[18] Patel A.K., Joshi D., Khan A., Jaisval G.K., Kumar A., Pathania R., Hasan W., Kushwaha T.N. (2024). Biology, diversity, distribution, and characterization of *Brevicoryne brassicae* (L.) cabbage. Int. J. Plant Soil Sci..

[19] Dharmasiri N., Kannangara S., Undugoda L., Munasinghe J., Madushika R., Thambugala K.M., Gunathunga C., Pavalakumar D. (2025). The Mycoremediation Potential of Phyllosphere Fungi in Urban Ornamental Plants in Sri Lanka with Mathematical Models for PAH Degradation. N. Z. J. Bot..

[20] Sari M.P., Wiyono S., Giyanto, Maharijaya A., Wahyuno D. (2025). Optimization of surface sterilization techniques to enhance the diversity of leaf endophytic fungal isolates from bitter ginger. IOP Conf. Ser. Earth Environ. Sci..

[21] Flegel T.W. (1980). Semipermanent Microscope Slides of Microfungi Using a Sticky Tape Technique. Can. J. Microbiol..

[22] Domsch K.H., Gams W., Anderson T.H. (1980). Compendium of Soil Fungi.

[23] Undugoda L.J.S., Kannangara S., Sirisena D.M. (2016). Aromatic Hydrocarbon-Degrading Fungi Inhabiting the Phyllosphere of Ornamental Plants on Roadsides of Urban Areas in Sri Lanka. J. Bioremediat. Biodegrad..

[24] Al-Nasir F., Hijazin T.J., Al-Alawi M.M., Jiries A., Al-Madanat O.Y., Mayyas A., Al-Dalain S.A., Al-Dmour R., Alahmad A., Batarseh M.I. (2022). Accumulation, Source Identification, and Cancer Risk Assessment of Polycyclic Aromatic Hydrocarbons (PAHs) in Different Jordanian Vegetables. Toxics.

[25] Amarasekara R.C.J., Kannangara B.T.S.D.P., Undugoda L.J.S. (2024). Bioremediation potential of leaf endophytic fungi in *Allium ampeloprasum* and *Brassica oleracea* var. *capitata*. Proceedings of the International Conference on Applied and Pure Sciences (ICAPS), Kelaniya, Sri Lanka, 11 October 2024.

[26] Fan Y., Ma Z., Zhang Y., Wang Y., Ding Y., Wang C., Cao S. (2022). Sulfur-Containing Compounds from Endophytic Fungi: Sources, Structures and Bioactivities. J. Fungi.

[27] Nicoletti R. (2021). Occurrence and Functions of Endophytic Fungi in Crop Species. Agriculture.

[28] Poveda J., Díaz-González S., Díaz-Urbano M., Velasco P., Sacristán S. (2022). Fungal Endophytes of Brassicaceae: Molecular Interactions and Crop Benefits. Front. Plant Sci..

[29] Poveda J., Zabalgogeazcoa I., Soengas P., Rodríguez V.M., Cartea M.E., Abilleira R., Velasco P. (2020). *Brassica oleracea* var. *acephala* (Kale) Improvement by Biological Activity of Root Endophytic Fungi. Sci. Rep..

[30] Padmajani M.T., Aheeyar M.M.M., Bandara M.A.C.S. (2014). Assessment of Pesticide Usage in Up-Country Vegetable Farming in Sri Lanka.

[31] De Lima Brossi M.J., Jiménez D.J., Cortes-Tolalpa L., van Elsas J.D. (2016). Soil-Derived Microbial Consortia Enriched with Different Plant Biomass Reveal Distinct Players Acting in Lignocellulose Degradation. Microb. Ecol..

[32] Zhang G., Wang J., Zhao H., Liu J., Ling W. (2021). PAH Degradation and Gene Abundance in Soils and Vegetables Inoculated with PAH-Degrading Endophytic Bacteria. Appl. Soil Ecol..

[33] Christian N., Sullivan C., Visser N.D., Clay K. (2016). Plant Host and Geographic Location Drive Endophyte Community Composition in the Face of Perturbation. Microb. Ecol..

[34] Anwar W., Shahid A.A., Haider M.S. (2017). Entomopathogenic Fungi: Introduction, History, Classification, Infection Mechanism, Enzymes, and Toxins. Biopesticides and Bioagents.

[35] Steffen K.T. (2003). Degradation of Recalcitrant Biopolymers and Polycyclic Aromatic Hydrocarbons by Litter-Decomposing Basidiomycetous Fungi. Ph.D. Thesis.

[36] Peng M., Aguilar-Pontes M.V., Hainaut M., Henrissat B., Hildén K., Mäkelä M.R., de Vries R.P. (2018). Comparative Analysis of Basidiomycete Transcriptomes Reveals a Core Set of Expressed Genes Encoding Plant Biomass Degrading Enzymes. Fungal Genet. Biol..

[37] Thion C., Cébron A., Beguiristain T., Leyval C. (2012). Long-Term In Situ Dynamics of the Fungal Communities in a Multi-Contaminated Soil Are Mainly Driven by Plants. FEMS Microbiol. Ecol..

[38] Thion C., Cébron A., Beguiristain T., Leyval C. (2013). Inoculation of PAH-Degrading Strains of *Fusarium solani* and *Arthrobacter oxydans* in Rhizospheric Sand and Soil Microcosms: Microbial Interactions and PAH Dissipation. Biodegradation.

[39] Pozdnyakova N., Muratova A., Bondarenkova A., Turkovskaya O. (2023). Degradation of a Model Mixture of PAHs by Bacterial–Fungal Co-Cultures. Front. Biosci. (Elite Ed.).

[40] Crittenden J., Raudabaugh D., Gunsch C.K. (2025). Isolation, Characterization, and Mycostimulation of Fungi for the Degradation of Polycyclic Aromatic Hydrocarbons at a Superfund Site. Biodegradation.

[41] Zafra G., Cortés-Espinosa D.V. (2015). Biodegradation of Polycyclic Aromatic Hydrocarbons by *Trichoderma* Species: A Mini Review. Environ. Sci. Pollut. Res..

[42] Escudero-Leyva E., Alfaro-Vargas P., Muñoz-Arrieta R., Charpentier-Alfaro C., Granados-Montero M.D.M., Valverde-Madrigal K.S., Pérez-Villanueva M., Méndez-Rivera M., Rodríguez-Rodríguez C.E., Chaverri P. (2022). Tolerance and Biological Removal of Fungicides by *Trichoderma* Species Isolated from the Endosphere of Wild Rubiaceae Plants. Front. Agron..

