# Supplementary material for: Bioremediation Potential of Leaf Endophytic Fungi in Allium ampeloprasum and Brassica oleracea var. capitata
Source: J Fungi (Basel). 2026 Apr 20;12(4):295. doi: 10.3390/jof12040295 (PMC13117999; doi:10.3390/jof12040295)
Supplement: Supplementary file 1 [file jof-12-00295-s001.zip › jof-4184293-supplementary.pdf]

Supplementary Table 1. Environmental conditions and site coordination details of sample collection area.

Site	Coordinates (Latitude, Longitude)	Elevation (m)	Soil Type	Avg. Temperature (°C)	Avg. Rainfall (mm)
Nuwara Eliya Town	6°57'43.09" N, 80°46'00.36" E	1896.78	Red Yellow Podzolic	18	7
Nanu Oya	6°56'30.22" N, 80°44'39.27" E	1635.22	Red Yellow Podzolic	20	6
St. Clair’s	6°56'47.99" N, 80°39'01.28" E	1217.38	Red Yellow Podzolic	20	7
Meepilimana (Control Site)	6°55'58.19" N, 80°47'07.90" E	1923.14	Red Yellow Podzolic	19	6

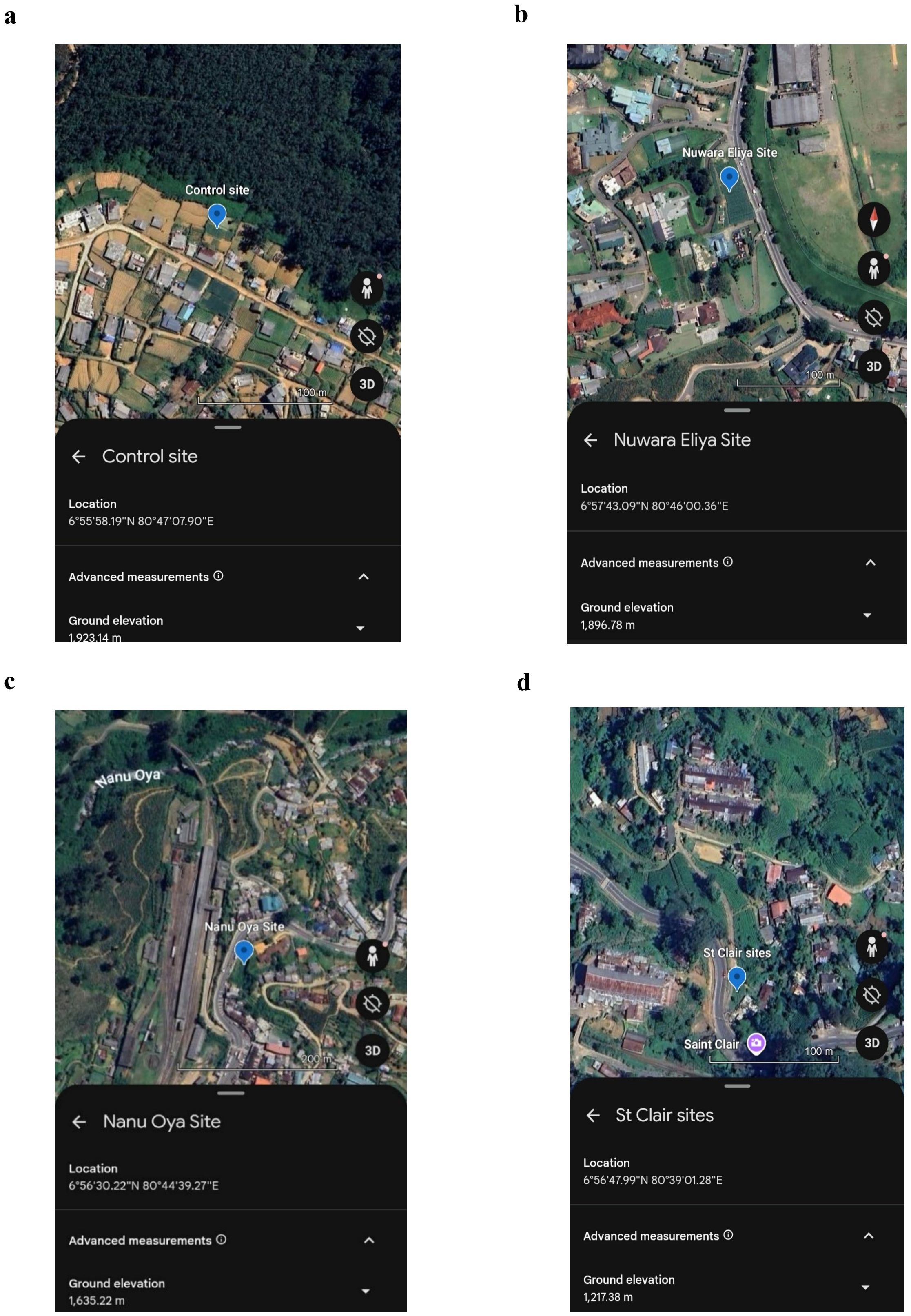

Supplementary Figure 1. Map coordinates of selected sample collection sites in Nuwara Eliya district (a) control site – Meeplimana, (b) Nuwara Eliya town, (c) Nanu Oya, (d) St Clair's

PAH standard curves for HPLC analysis and relevant R^2 values

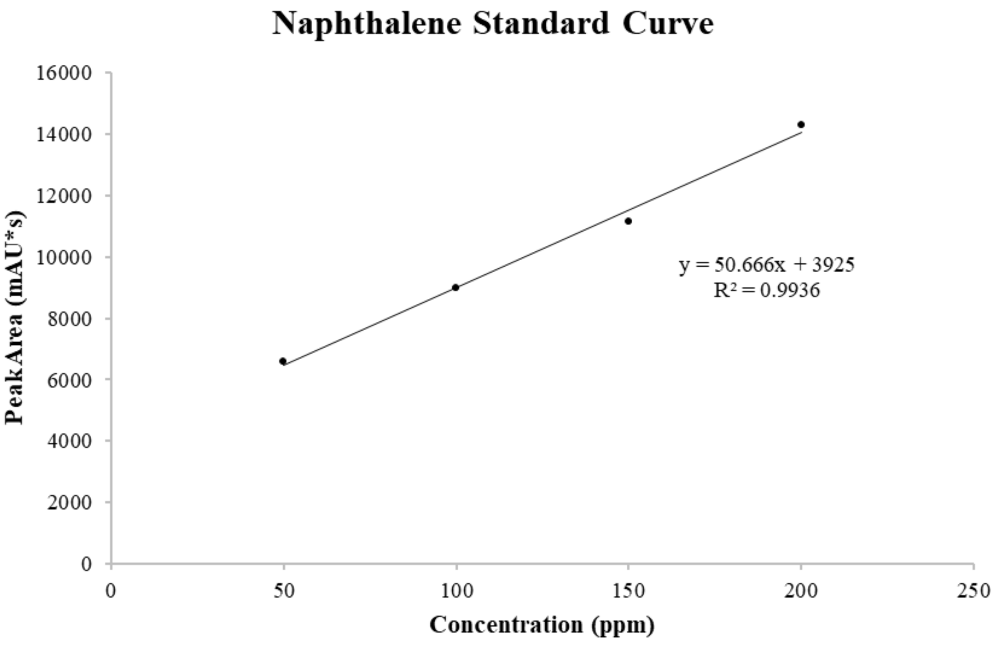

Supplementary Figure 2.1. Naphthalene standard curve

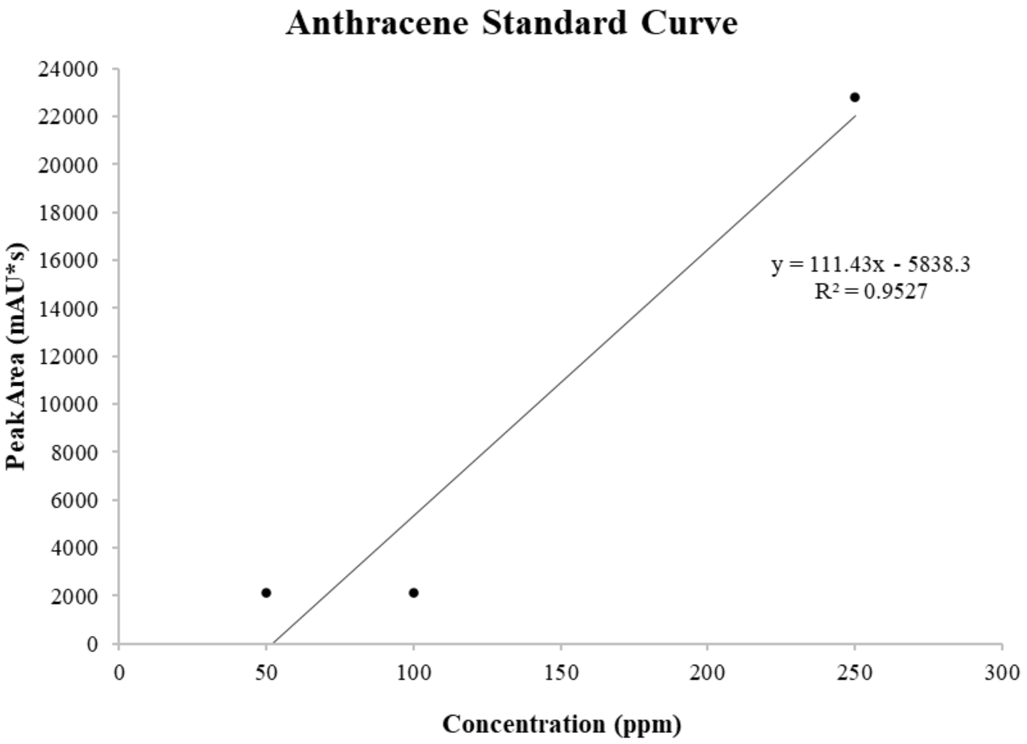

Supplementary Figure 2.2. Anthracene standard curve

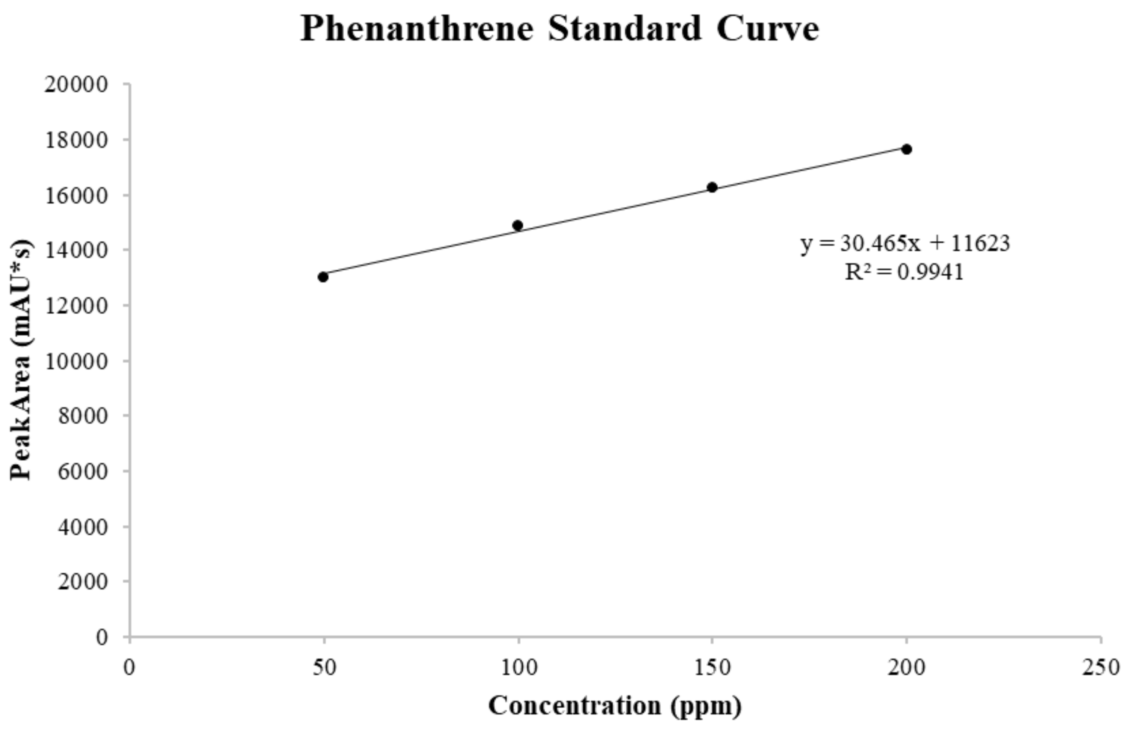

Supplementary Figure 2.3. Phenanthrene standard curve

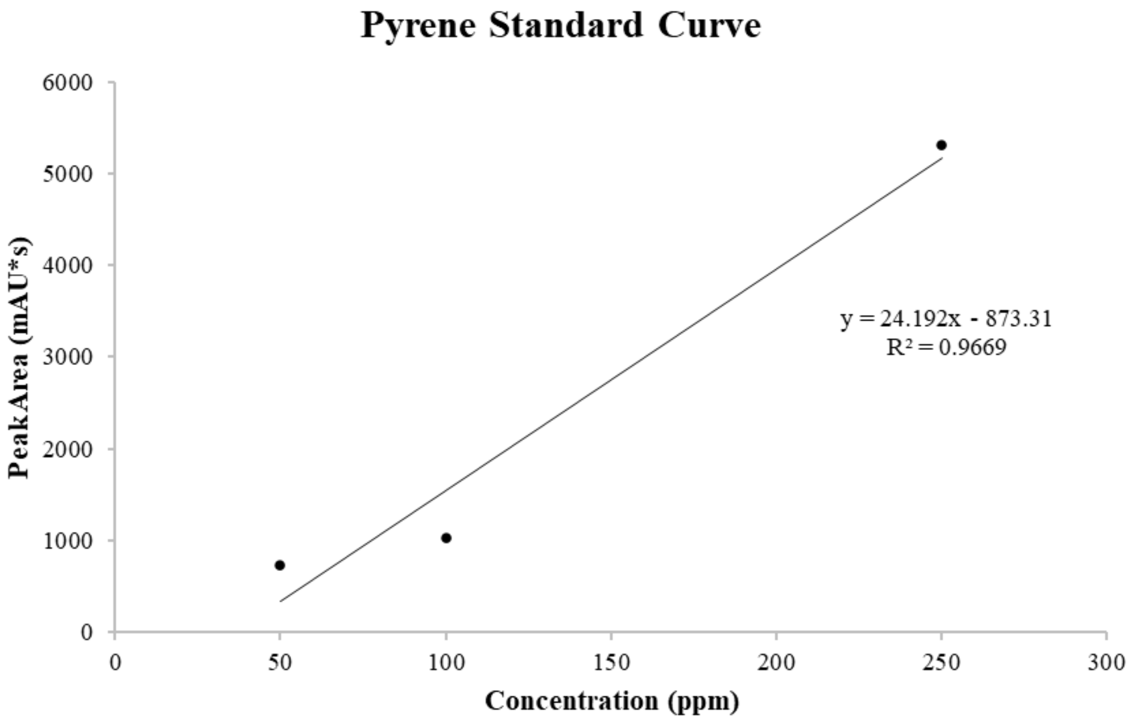

Supplementary Figure 2.4. Pyrene standard curve
